# Advancement in Gynæcology

**Published:** 1880-02

**Authors:** J. G. Westmoreland

**Affiliations:** Atlanta, Ga.


ATLANTA
Medical and Surgical Journal.

VOL. XVII.] FEBRUARY—1880. [No. 11.

### Original Communications.

### ADVANCEMENT IN GYNÆCOLOGY.

BY J. G. WESTMORELAND, M.D., ATLANTA, GA.

Among the many changes in medicine which have occurred in the last quarter of a century, none are more striking than that found in the treatment of uterine affections. Thirty years ago medical men were content in the treatment of uterine diseases by affording relief from the consequent reflex nervous troubles called "hysterics," and an attempt to prevent their return by "nervous tonics" and "emmenagogues." Many did not even attribute the nervous perturbation to uterine or other local origin, but to primary "general nervous debility," and hence "anti-spasmodics," stimulants and nervous tonics were relied on for present and prospective freedom from the paroxysms. At this period, it is true, certain organic symptoms or results of diseased uterus were recognized, and sometimes treated by special as well as general means. Leucorrhœa and prolapsus were then the prevailing uterine diseases (?), and treated by the introduction of globular, oval or round pessaries composed of glass, wood or metal for the latter, and astringent vaginal injections for the former. Females are now to be found, not a few, who may be pointed to as sterile monuments of this unsuccessful course of procedure. Dysmenorrœa and amenorrhœa were also treated by name as radical diseases, without regard to the pathological conditions leading to these forms of functional disturbance, whether scanty, retained, suppressed, painful or excessive.

We would not be understood as saying that no member of the profession took a more rational view of this subject, but that the general practitioner prescribed thus emperically, without any settled views of uterine pathology.

The first step of advancement in this period of empirical gynæcology, was that of taking observation, through a cylindrical speculum, of so-called ulcers of the os uteri, in the form of abrasions, and their treatment by the application of lunar caustic. When not too violently and frequently cauterized, relief was afforded to this unimportant trouble, the only lesion revealed to the inexperienced observer; and when he was so fortunate as not to increase the difficulty, great was his surprise to find that with a smooth and apparently healthy os, the female, after being "discharged cured," still suffered from constitutional nervous disturbance, which, though improved, was still far from being relieved. The reflex or hysterical nervous troubles still recurring when no prolapsus, disturbed menstruation or ulcers remained, was a matter of great astonishment, and the case being considered one of primary general nervous debility, the patient was drugged and tortured for months with every conceivable feruginous and other nervous tonic and stimulant.

Finally, Scanzoni and others saw something beyond the ulcerated os to acconnt for continuance of the various reflex pains and aches. It was observed that the tender and painful womb, often of increased size and greater specific gravity, discharged constantly from the os an albuminous, tenacious, transparent fluid. These discoveries led to thorough investigation on this subject, which resulted in the establishment of facts connected with organic lesions explanatory of all the symptoms above enumerated. It was ascertained that engorgement of the womb gave not only increased actual weight and size of the organ, but much greater specific gravity. This state precedes and tends to the production of prolapsus and other varieties of displacement, intersticial hypertrophy, chronic inflammation of various tissues connected with the uterus and its appendages, and ulceration of the os and cervix. The surrounding cellular tissue may be the subject of this inflammation known as areola or peri-uterine hyperplasia, and when in a state of inordinate engorgement with hemorrhagic tendency, is called hæmatocele, developing abscesses and bloody tumors in the pelvis. The uterine walls, including the Fallopian tubes and ovaries, take on this inflammatory state, giving the tenderness and pain so frequently experienced in these parts. Particularly prone to this condition is the intra-uterine mucous membrane; and the opinion of some authors to the contrary notwithstanding, chronic endo-metritis is as often, if not more frequently, met with than any other form of uterine disease. It exists, not only in connection with the same condition in the walls of the uterus, but often where no evidence of inflammation is found in any other part of the organ. The importance of the internal surface as a point for the application of remedies is manifested in the relief of soreness and pain of the body of the womb and its appendages by local treatment directed to the internal mucous membrane alone.

The use of passaries has not increased with the advancement in gynecology. Whereas, they once constituted almost the sole treatment in some cases, they are applied now, not with the view of producing direct curative effect, but to allow the necessary amount of exercise, which in some instances cannot be had without support to the prolapsed uterus. All must admit, however, that without this compensating benefit, the mechanical irritation should by all means be avoided in chronic metritis. External abdominal support by a spring supporter, relieving the womb from pressure of the abdominal viscera, will generally be sufficient, and if so, is decidedly preferable. This should be used by females, the subject of diseased uterus, whether perceptible displacement of any kind exists or not; for prolapsus and other forms are produced by engorgement, rritation or inflammation, and the malpositions always aggravate the existing disease. Supports are intended to give only temporary relief, as it is believed, a permanent normal position of the uterus cannot certainly be secured without restoration of the organ to healthy condition.

Various forms of pessaries have been invented and recommended, but all, to some extent, are subject to the above objection. Notwithstanding this, gynecologists think proper to use them occasionally.

So much for advancement in our knowledge of the true cause and proper treatment of uterine displacements.

The modes of treating organic disease which leads to prolapsus, leucorrhœa, and the various forms of menstrual derangement are constantly becoming more and more perfect.

Different practitioners have their favorite modes of accomplishing the same object. For example, vascular engorgement of the womb, which precedes and accompanies most of the uterine troubles, is relieved by one with scarification and puncture of the os, by another with leeches, while others content themselves with the depleting or detergent effect of cottonwool saturated with glycerine and applied to the os. Still more numerous are the medicinal agents and modes of application for the catheretic or local alterative action necessary to the permanent relief of chronic metritis and endometritis. Nitrate of silver, sulph. copper, iodine, zinc, carbolic, nitric and cromic acids, have each its advocate. These are introduced into the cavity in liquid form by saturated lint, sponge, cotton, or cloth tent; or injected into the cavity with a syringe made for the purpose. Discussions arise as to the probable destruction of sound structures by some of these and the consequent deformity by the formation of cicatricial tissue. All must admit that fuming nitric acid will have this effect, and stenosis may be the result of its action upon the cervical canal. None, however, who have made the test with carbolic acid will attribute to it this degree of action. While pain and even excorition may be the result of its escape and contact with the pudendum, it may be injected without dilution into the uterus with no more pain than other substances used for the purpose, and without destruction of sound tissue.

The writer desires to call attention also to the important fact that no remedy in use has been found equal to this in the local treatment of cancerous ulceration. Applied to the ulcer in full strength, it is neither painful nor unnecessarily destructive to tissue. Indeed, pain and irritability are relieved by it. What effect the remedy may have on the cancerous diathesis, if such exist, of course has not been determined, but that it will correct the condition of maglignant ulcer, no one will doubt who has made thorough application of crystalized carbolic acid warmed to the liquid state. Used in this way, cancerous ulcer of the os, neck and vigina has been known kindly to improve. Being also one of our best antiseptics, the very offensive odor attending uterine cancer is promptiy destroyed by it. The application every third or fourth day by touching with saturated cloth or sponge will give gratifying results to any physician who has the charge of such unpleasant and dangerous disease.

